# Interpersonal Dynamics at Work: How Positive and Negative Experiences Simultaneously Influence Work Attitudes

**DOI:** 10.3390/bs16010156

**Published:** 2026-01-22

**Authors:** Madison A. Malcore, Declan O. Gilmer, Vicki J. Magley

**Affiliations:** 1 Department of Psychological Sciences, University of Connecticut, Storrs, CT 06269, USA; 2 The Clearing, Inc., Washington, DC 20036, USA

**Keywords:** incivility, prosocial experiences, interpersonal experiences, job attitudes, job stress

## Abstract

Workplace mistreatment and positive interpersonal experiences are not often considered simultaneously in empirical research. However, people are realistically experiencing positive and negative interpersonal experiences at work regularly. The goal of this study is to fill this gap by examining the relative importance of both incivility and prosocial experiences on people’s job attitudes. Data from a large university in the northeastern United States revealed significant relationships between incivility and prosocial experiences and cynicism towards organizational change, job satisfaction, turnover intentions, and affective commitment. Further, relative weights analysis, controlling for established job stressors, identified interpersonal experiences as accounting for about half of the variance in job attitudes. This demonstrates the strong role that these experiences have in shaping attitudes. Further, experiences coming from supervisors were highlighted as particularly important. Follow-up analyses provide preliminary evidence that these interpersonal experiences have a stronger influence on job attitudes for racial minority workers than for white workers. Implications and future directions are discussed.

## 1. Introduction

Workplace interpersonal experiences are complex. Working with people inherently results in a mix of positive and negative experiences. One important form of negative interpersonal experience is workplace incivility, or low-level mistreatment with an ambiguous intent to harm (Andersson & Pearson, 1999). This type of mistreatment is relatively common, with 90% of workers reporting incivility in their working life (Porath & Pearson, 2013). Incivility experiences harm targets’ well-being (e.g., Grandey et al., 2022; Magley et al., in press) as well as job attitudes (Chris et al., 2022). Further, positive experiences at work, particularly in relation to leaders, improve job attitudes (Gao et al., 2022). Therefore, it has been demonstrated that these interpersonal experiences are critical for employee well-being and their commitment to their organizations.

A limitation of the current literature is that positive and negative interpersonal experiences are studied in isolation from one another. Practically, we know that working relationships are not this simplistic. An employee regularly experiences both mistreatment and prosocial experiences, which come from different or even the same source in their working lives. Given a recent call to study how positive and negative relationships may simultaneously affect work attitudes (Judge et al., 2017), an investigation of the complex interplay of interpersonal experiences is warranted. The current study aims to examine the influence of both positive and negative experiences on job attitudes when examined in tandem. We turn next to a discussion of the types of experiences that employees frequently have.

### 1.1. Workplace Incivility

Workplace incivility behaviors involve ambiguous behaviors that have an unclear intent to harm the target (Andersson & Pearson, 1999). Workplace incivility is a common experience among workers, with between 60% and 80% of individuals reporting experiencing workplace incivility within the past five years (Cortina et al., 2001). Other literature has found that 98% of workers report experiencing incivility at least once in their work tenure (Porath & Pearson, 2013). Although this type of mistreatment is subtle, the experience of workplace incivility has consistently been shown to have negative effects on targets.

It is well-established that negative work relationships can be stressful (e.g., Grandey et al., 2022) and lead to increased negative emotions (Sakurai & Jex, 2012). These experiences and subsequent stress and negative emotions have been demonstrated to have detrimental effects on targets’ job attitudes. Specifically, the field has documented negative associations between interpersonal mistreatment and affective commitment (Chris et al., 2022), intentions to leave the organization (Chris et al., 2022; Cortina et al., 2013), organizational cynicism (Manzoor et al., 2020), and job satisfaction (Chris et al., 2022; Loh et al., 2021). This suggests that there is ample evidence to support the harm that workplace incivility has when examined independently of other forms of interpersonal experiences; however, what remains unknown is if and how incivility interacts with prosocial experiences to shape job attitudes.

### 1.2. Prosocial Experiences

Prosocial behaviors are defined as voluntary actions intended to benefit another person (Eisenberg & Spinrad, 2014). Within the workplace, these behaviors may be enacted by individuals, groups, or the organization as a whole, with the intention to promote or protect the welfare of others (e.g., colleagues, teams, or the broader organization; Bolino & Grant, 2016; Brief & Motowidlo, 1986; Hart, 2024). Prosocial behavior covers a broad range of actions, including assisting co-workers with job-related and personal matters (Brief & Motowidlo, 1986). The main positive experiences associated with work relationships include social support, companionship, leadership-specific variables (e.g., leader-member exchange; trust), mentorship, and civility (Stephens et al., 2012). In the context of this study, we will be exploring low-intensity prosocial experiences, such as being the target of genuine concern, courtesy, or interest, receiving help on work-related problems, and noticing and complimenting work-related accomplishments. This ensures that the intensity of the prosocial and incivility experiences is roughly equal, making comparisons between their impacts clear and easy to interpret.

Positive organizational scholars have shown that workplace relationships can also be pleasant and resource-generating (Stephens et al., 2012). This is supported by the broaden and build theory (Fredrickson, 2001), which was developed to explain the influence positive emotions have on important psychological states. This theory posits that positive emotions encourage exploration, resulting in the building of psychological, intellectual, and social resources (Fredrickson, 2001). This theory has been considered to be cyclical, wherein broadened resources developed through previous positive emotions result in improved coping during adversity (e.g., experiencing mistreatment; Fredrickson, 2001). In this way, positive experiences may act as a self-reinforcing feedback loop wherein effective coping strategies and emotional resources are strengthened over time. This is supported by empirical findings that having positive interpersonal workplace experiences increases feelings of social bonds in the workplace, which increases feelings of engagement (O’Connell et al., 2018). Additionally, these positive experiences are also related to decreased exhaustion and work disengagement (Kersten et al., 2022). Overall, these findings suggest that prosocial experiences may improve targets’ work attitudes by increasing positive emotions, increasing work engagement, and strengthening socioemotional ties to their working environment.

Additional evidence for the relationship between prosocial experiences and job attitudes has been empirically tested. For instance, the positive qualities of working relationships have been linked to higher levels of affective commitment (Gao et al., 2022). Further, having workplace friendships that fulfill prosocial roles, such as social support and helping behavior, is associated with increased affective commitment (Y.-C. Chen et al., 2024). Finally, when relationships between supervisors and coworkers are reported as more positive, people report increased job satisfaction (Poljašević et al., 2021). Compared to incivility, prosocial experiences have been less empirically explored. This is partially due to the focus of inquiry being on enacting prosocial behaviors, rather than experiencing them (Dastgheib et al., under review). While less empirically explored than workplace mistreatment, there is evidence to suggest that prosocial workplace experience will directly influence job attitudes.

### 1.3. The Intersection of Positive and Negative Experiences

Work relationships exist on a spectrum from negative to positive (Methot et al., 2017), depending on their context (e.g., workplace climate), content (e.g., positive and negative experiences), and appraisal. The meaning employees make out of positive and negative experiences may sometimes be straightforward; that is, negative experiences can lead to more negative attitudes toward their coworkers or their job. However, when considering the full spectrum of interpersonal experiences both across and within social relationships, the interpretation of experiences and their impacts may become more complex. Moreover, consider the impact of a negative experience with one individual versus multiple individuals in one’s network. Overall, the combination and nature of interpersonal experiences is complex and may, in turn, have complex associations with organizational attachment.

Literature supports the notion that negative interpersonal experiences at work can be harmful (e.g., Grandey et al., 2022), and there is also some preliminary empirical evidence that supports the notion that positive interpersonal experiences at work can be helpful (e.g., Stephens et al., 2012). However, these literatures are not well integrated. Thus, the current study focuses on the attitudinal outcomes of various person-level combinations of positive and negative work experiences. Specifically, this study focuses on the effect of interpersonal experiences—both positive and negative—on cynicism toward organizational change, job satisfaction, turnover intentions, and affective commitment.

### 1.4. Job Attitudes

Cynicism toward organizational change refers to employees’ perceptions that organizational change efforts will be unsuccessful, and change actors are disinterested or ineffective (Reichers et al., 1997). This cynicism is often directed at efforts to improve organizational functioning or working conditions for employees. In the higher education context, cynicism toward organizational change is strongly related to information quality, social influence, and trust in management (Qian & Daniels, 2008). Workplace mistreatment, specifically ostracism, is linked to increased cynicism toward organizational change (Sahoo et al., 2023). Meta-analytic work has also identified group cohesion and cynicism toward colleagues as sources of cynicism toward organizational change (Choi, 2011). Therefore, cynicism toward organizational change is at least partially rooted in interpersonal experiences at work, in particular with management.

In creating the Job Descriptive Index measure of job satisfaction, Smith and colleagues refer to job satisfaction as “persistent feelings toward discriminable aspects of the job situation” (Smith et al., 1969, p. 37). Job satisfaction literature notes that job satisfaction contains both cognitive and affective components. Job satisfaction may be particularly relevant to the examination of interpersonal experiences because they elicit proximal affective responses, which may, in turn, predict job satisfaction. As an example, meta-analytic evidence shows that coworker social support has a moderate positive association with job satisfaction, and that this association was stronger for affective support (ρ = 0.402) compared to instrumental support (ρ = 0.280; Chiaburu & Harrison, 2008).

Although employees leave organizations for various reasons (Hom et al., 2017), turnover intentions are one of the most proximal predictors of actual turnover. A recent McKinsey & Company report noted that there is a clear disconnect between employees’ cited reasons for quitting and employers’ assumptions about why employees quit (De Smet et al., 2021). According to the report, employees strongly valued a sense of belonging and supervisory relationships, but employers rated these variables as less important. Conversely, it is no surprise that negative relationships are associated with increased turnover intentions and actual turnover. For example, a recent meta-analysis with 46 primary studies found an average correlation of r = 0.31 between experienced incivility and turnover intentions (Namin et al., 2021).

Finally, organizational commitment can be broken down into affective (desire to stay), normative (obligation to stay), and continuance (need to stay) commitment (Meyer & Allen, 1991). Affective commitment is the form of commitment typically most desirable to organizations, because it indicates that employees remain in the organization simply because they want to stay. Regarding interpersonal experiences, scholars argue that supervisor behaviors can have a strong impact on perceived organizational support (Rhoades & Eisenberger, 2002) and, in turn, affective commitment (Kabins et al., 2016). Meta-analytic evidence also shows that interpersonal experiences with coworkers, both positive (ρ = 0.317) and negative (ρ = −0.250), are associated with general organizational commitment (Chiaburu & Harrison, 2008). Overall, the literature suggests that employees with positive interpersonal experiences tend to have higher affective commitment (Rousseau & Aubé, 2010) and that positive experiences may buffer against the effects of simultaneous negative experiences (e.g., incivility; Anwar & Sidin, 2016).

### 1.5. The Current Study

The current study aims to understand how incivility and prosocial experiences at work influence job attitudes, both independently and when considered in tandem with one another. Additionally, evidence suggests that there are potential differences in the impact of mistreatment experiences with supervisors versus with peers. Hitlan and Noel (2009) identified that workplace exclusion and ostracism are associated with unique outcomes when coming from supervisors, compared to coworkers. Specifically, when ostracized by coworkers, people are more likely to engage in interpersonal counterproductive work behaviors; however, when ostracism comes from management, counterproductive behaviors target the organization (Hitlan & Noel, 2009). Furthermore, meta-analytic evidence supports that supervisor-perpetrated mistreatment may be more detrimental to job attitudes than coworker-perpetrated mistreatment (e.g., Hershcovis & Barling, 2010). Presumably, this relationship will extend to prosocial experiences as well due to the potential for increased organizational knowledge, the ability to provide more tangible support, and the weight of supervisor validation or compliments. This is demonstrated by the strong link between positive supervisor relationships and perceived organizational support (Rhoades & Eisenberger, 2002). Therefore, this study will examine the role of coworker and supervisor experiences as unique variables. Finally, this study will include general job stressors (role ambiguity, role overload, and general job stress) as covariates to identify the degree to which interpersonal experiences shape job attitudes in relation to constructs that have been well-established in the job stress literature.

Therefore, the current study will test several hypotheses related to the direct effects of interpersonal experiences at work, followed by research questions exploring how these experiences influence attitudes when examined simultaneously. First, workplace incivility has meta-analytic evidence to support its effect on affective commitment, intentions to leave the organization, and job satisfaction (Chris et al., 2022). Further, initial empirical support has been provided for incivility increasing targets’ organizational cynicism, broadly (Manzoor et al., 2020). This study aims to extend this finding to the specific dimensions of cynicism toward organizational change.

**Hypothesis** **1.** *Supervisor (H1a) and Coworker (H1b) incivility experiences will be associated with increased cynicism toward organizational change.*

**Hypothesis** **2.** *Supervisor (H2a) and Coworker (H2b) incivility experiences will be associated with decreased job satisfaction.*

**Hypothesis** **3.** *Supervisor (H3a) and Coworker (H3b) incivility experiences will be associated with increased turnover intentions.*

**Hypothesis** **4.** *Supervisor (H4a) and Coworker (H4b) incivility experiences will be associated with decreased affective commitment.*

Prosocial experiences have received less attention in the organizational literature, but theoretical frameworks, such as the broaden and build theory (Fredrickson, 2001), support the assertion that these experiences will shape job attitudes through increased positive emotions and resources. Additionally, initial empirical evidence provides support for the influence of positive relationships and experiences on affective commitment (Y.-C. Chen et al., 2024; Gao et al., 2022) and job satisfaction (Poljašević et al., 2021). Therefore, the current study aims to test relationships between low-level prosocial experiences (e.g., being the target of genuine concern, courtesy, or interest, receiving help on work-related problems, and noticing and complimenting work-related accomplishments) and job attitudes.

**Hypothesis** **5.** *Supervisor (H5a) and Coworker (H5b) prosocial experiences will be associated with decreased cynicism toward organizational change.*

**Hypothesis** **6.** *Supervisor (H6a) and Coworker (H6b) prosocial experiences will be associated with increased job satisfaction.*

**Hypothesis** **7.** *Supervisor (H7a) and Coworker (H7b) prosocial experiences will be associated with decreased turnover intentions.*

**Hypothesis** **8.** *Supervisor (H8a) and Coworker (H8b) prosocial experiences will be associated with increased affective commitment.*

This study will also explore how considering these experiences simultaneously will shape job attitudes. The complex nature of everyday workplace interpersonal experiences has been largely overlooked in empirical research; however, there have been calls to examine the full breadth of workplace experiences simultaneously to gain a clearer understanding of the nature of true interpersonal experiences at work (Judge et al., 2017). This study aims to achieve this by examining the unique effects of incivility and prosocial experiences on job attitudes.

Research Question: While controlling for established job stressors, how much unique variance is accounted for by coworker and supervisor incivility and prosocial experiences on cynicism toward organizational change (RQa), job satisfaction (RQb), turnover intentions (RQc), and affective commitment (RQd).

## 2. Method

### 2.1. Participants and Survey Information

The sample used to test these hypotheses is an intraorganizational archival dataset. Data were collected from university employees at a large northeastern university in 2017. Responses were collected from academic departments as well as operations and service staff. A total of 4934 faculty and staff and 2089 graduate assistants were invited to take the survey. Of the 4934 faculty and staff employees invited, approximately 37% took the survey. Of this 37%, surveys were omitted for 242 who did not complete at least 50% of the survey, rendering a usable sample from approximately 35% (*n* = 1725 employees). The average age of respondents was 43.24 years. This sample was primarily comprised of individuals who identified as women (*n* = 960; 55.7%). Additionally, 33.8% of the sample identified as men (*n* = 583), and two individuals identified as non-binary (0.1%). Ten respondents indicated that their gender did not fit within the provided options, 71 indicated that they preferred not to disclose their gender, and 99 respondents did not answer this question. In terms of race/ethnicity, the majority of respondents indicated that they are white (*n* = 1449; 84%). Thirty-nine participants (2.3%) identified as Black, 66 (3.8%) as Hispanic or Latino, 56 (3.2%) as Asian or Pacific Islander, 6 (0.3%) identified as Native American or Alaskan, and 68 (3.9%) indicated that they identified with a race/ethnicity not listed. One-hundred and thirty-one survey respondents did not provide information regarding their race and ethnicity. Of the 1157 respondents who indicated their work unit, 57% percent of survey respondents were faculty members, with the remaining datapoints coming from university staff. On average, respondents have worked for the university for 11.45 years and in their current positions for 8.34 years.

### 2.2. Measures

Interpersonal Experiences. Experienced workplace incivility was measured using a composite scale containing items from the General Incivility Scale (Cortina et al., 2001) as well as a scale measuring experienced exclusion (Martin & Hine, 2005). This scale asked respondents to identify how frequently they experienced incivility/exclusion over the past year by their supervisors and coworkers in the past year. Separate scores were computed for coworker and supervisor incivility. Response options were measured on a 5-point frequency scale (0 = never to 4 = many times). Four items on this scale measured experiences of general incivility (e.g., receiving hostile looks, stares, or sneers; having jokes made at their expense; being addressed inappropriately or unprofessionally). Two items on this scale measured respondents’ experiences of exclusionary behavior: “did not consult you in reference to a decision you should have been involved in” and “intentionally failed to pass on information which you should have been made aware of.” Exploratory factor analysis was conducted to identify the appropriateness of combining incivility and exclusionary mistreatment items. Findings suggest a single-factor solution accounts for 62.1% and 60.72% of variance for supervisor and coworker scales, respectively. Further, all factor loadings were equal to or above 0.70. Therefore, combining these items is statistically supported in these data. Scores were aggregated for supervisor and coworker experiences separately.

Prosocial experiences were also measured on a 5-point frequency scale (0 = never to 4 = many times). Respondents were asked to consider how frequently they have experienced prosocial behaviors from their coworkers and supervisors in the past year. Items were developed to reflect the same valence as incivility experiences (Dastgheib et al., under review). Specifically, items were meant to be low-intensity, everyday prosocial behaviors that captured helping, inclusion, and acknowledgment/appreciation of one’s work. Respondents were asked to consider the frequency of their experiences with their supervisors and coworkers in the past year. Items include “showed you genuine concern and courtesy,” “went out of his or her way to help you with a work-related problem,” “helped you do your job to the best of your ability,” “noticed when you did your best possible work,” expressed interest in your work-related opinions,” and “complemented you on your work accomplishments.” Separate scores were computed for coworker and supervisor experiences.

Stress Covariates. Common occupational stressors were utilized as covariates to determine the amount of variance accounted for by interpersonal behaviors beyond traditional workplace stressors. Due to the archival nature of this dataset, abbreviated scales on established job stressors were utilized. Role ambiguity was measured using three items adapted from Rizzo et al. (1970): “I feel certain about how much authority I have,” “I know what my responsibilities are,” and “I know exactly what is expected of me.” All items were reverse-coded. Role overload was measured using a single item: “the amount of work I am asked to do is fair” (Schaubroeck et al., 1989). Both of these scales were measured on a 7-point Likert scale (1 = strongly disagree to 7 = strongly agree). Job stress was measured using a single item, “How would you rate your current stress level?” rated on a scale from 0 (“as bad as it can be”) to 10 (“as good as it can be;” Clark et al., 2011).

Outcomes. The effects of interpersonal experiences on common work attitudes were examined in this study. This section of the survey directed participants to consider aspects of their job at the organization. Cynicism toward organizational change was scored on four reverse-coded items (e.g., “Attempts to make things better at [the university] will produce good results”; “The people responsible for solving problems at [the university] try hard to solve them”). These items were selected and adapted for a university sample from Wilkerson et al. (2008) measure of cynicism toward organizational change. Job satisfaction was measured with a single item: “All in all, I am satisfied with my job” (Cammann et al., 1983). A single item was included for job satisfaction; however, this does not raise concerns about the outcomes of our analyses because a single-item measurement of job satisfaction has been empirically supported (e.g., Dolbier et al., 2005). Turnover intentions were measured using three items taken from Kelloway et al. (1999) scale. Items include “I think about quitting my job at [the university],” “I plan to look for a new job within the next year,” and “I have considered leaving [the university] for advancement opportunities not available here.” Affective commitment was measured with four items (Meyer et al., 1993), including “I feel a strong sense of belonging to [the university]” and “I would be very happy to spend the rest of my career at [the university].” Job self-efficacy was included as an outcome validity check. Job self-efficacy is not expected to be strongly influenced by interpersonal experiences, so we expected our results to reflect that. This scale included three items from G. Chen et al.’s (2004) scale: “I have no problem meeting the expectations that my employer has for me,” “I can successfully organize and prioritize my duties at work,” and “I am confident in my ability to meet most deadlines on my job.” All items were measured on a 7-point Likert scale of agreement (1 = strongly disagree to 7 = strongly agree).

## 3. Results

To evaluate the measurement quality, Cronbach’s Alphas were calculated, and all scales demonstrated good reliability (See Table 1). Independent samples *t*-tests were conducted to identify any meaningful differences between faculty and staff on outcome measures to ensure combining these groups for analytic purposes was appropriate. Findings suggest that there were no significant differences between the two group on job satisfaction (t(1145) = 0.28, *p* = 0.79), turnover intentions (t(1142) = 0.98, *p* = 0.33), affective commitment (t(1151) = −0.86, *p* = 0.39), and cynicism towards organizational change (t(1146) = 0.92, *p* = 0.36). Therefore, it was deemed appropriate to combine these groups hypothesis and research testing purposes.

Frequency analyses were run to identify how many respondents report positive and negative experiences at work. When examining how many participants reported at least one prosocial experience, 99.4% reported prosocial experiences with their supervisors, and 99.7% reported at least one prosocial experience with their coworkers. For incivility, 82.1% of respondents reported at least one incident of supervisor incivility, and 84.5% reported coworker incivility. Further, correlation analyses were performed to identify the relationship between variables in this study (see Table 1). To test the hypotheses, regression analyses were performed to identify the direct relationships between positive and negative experiences and job attitudes. Finally, relative weights analyses were performed to identify the relative importance of interpersonal experiences at work, answering the research question in this study. This statistical approach allows researchers to address multicollinearity in predictor variables when utilizing a regression framework.

### 3.1. Hypothesis Testing

To test the direct effects of each type of interpersonal experience on work attitudes, simple linear regression analyses with list-wise deletion were conducted in IBM SPSS (version 28). In support of Hypotheses 1–4, supervisor incivility had a significant relationship with cynicism toward organizational change (β = 0.36, t = 15.58, *p* < 0.001), job satisfaction (β = −0.44, t = −19.68, *p* < 0.001), turnover intentions (β = 0.39, t = 17.11, *p* < 0.001), and affective commitment (β = −0.36, t = −15.41, *p* < 0.001). Further, coworker incivility demonstrated significant relationships in the predicted direction with cynicism toward organizational change (β = 0.28, t = 11.44, *p* < 0.001), job satisfaction (β = −0.34, t = −14.54, *p* < 0.001), turnover intentions (β = 0.31, t = 12.99, *p* < 0.001), and affective commitment (β = −0.26, t = −10.67, *p* < 0.001). Therefore, incivility had a significant relationship with all job attitudes measured in this study in the anticipated direction, supporting study hypotheses.

When examining prosocial experiences, Hypotheses 5–8 were supported. Specifically, supervisor prosocial experiences were related to cynicism toward organizational change (β = −0.41, t = −18.01, *p* < 0.001), job satisfaction (β = 0.50, t = 23.21, *p* < 0.001), turnover intentions (β = −0.38, t = −16.30, *p* < 0.001), and affective commitment (β = 0.44, t = 19.67, *p* < 0.001). Coworker prosocial experiences were associated with lower cynicism toward organizational change (β = −0.32, t = −13.26, *p* < 0.001), higher job satisfaction (β = 0.35, t = 14.73, *p* < 0.001), lower turnover intentions (β = −0.26, t = −10.65, *p* < 0.001), and higher affective commitment (β = 0.36, t = 14.22, *p* < 0.001). Overall, these findings suggest that incivility and prosocial experiences play a significant role in shaping job attitudes.

### 3.2. Relative Weights Analysis

To test our research question in this study, relative weights analyses were conducted for each outcome of interest. This approach was selected because it addresses concerns of multicollinearity in predictor variables. As observed in our correlation analyses, interpersonal experiences relate to one another meaningfully. This may be partially due to the nature of job tasks being interdependent and requiring teamwork or communication. Further, we are breaking out incivility and prosocial experiences by coworkers and superiors; therefore, it may be the case that norms within a workgroup are informing the behaviors of both supervisors and coworkers, leading to a high correlation between these groups. Relative weights analysis identifies the unique percentage of total variance accounted for by all of our predictor variables in each criterion of interest.

Job self-efficacy is not expected to have a strong relationship with interpersonal experiences at work, and this was supported by the outcomes of this analysis, suggesting statistical validity. The overall regression model accounted for 32% of the variance in job self-efficacy. The strongest contributor to this variance is role ambiguity (59%), followed by role overload (25%) and job stress (10%). Coworker and supervisor positive and negative experiences each accounted for 2% of variance or less.

For each of our outcomes of interest, about half (48.8–54.97%) of the variance was accounted for by job stressors. This finding suggests that workplace mistreatment literature should not overlook the importance of these established job stressors as covariates, so that clear relationships between interpersonal experiences and job attitudes can be established. Full breakdowns of these job stressors can be found in Table 2.

**Figure 1:**
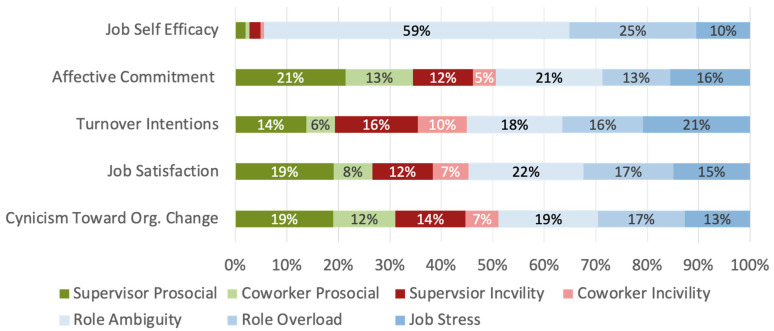
Visualization of Variance Breakdown for Total Sample.

Focusing on the unique contributions of interpersonal experiences, supervisor experiences, both prosocial and uncivil, were established as particularly important for shaping job attitudes. When cynicism toward organizational change was the outcome, the full model accounted for 29% of variance. Of that 29%, supervisor prosocial experiences accounted for 19% of variance, and supervisor incivility accounted for 14% of variance. Coworker prosocial and incivility experiences accounted for 13% and 7% of variance, respectively. For job satisfaction, supervisor prosocial experiences once again emerged as the strongest explanatory factor (19%), followed by supervisor incivility (12%), coworker prosocial experiences (8%), and coworker incivility (7%). Thirty percent of the variance in turnover intentions was accounted for by this model. Supervisor incivility explained the most variance in this model (16%), followed by supervisor prosocial experiences (14%), then coworker incivility (10%), and finally coworker prosocial experiences (6%). Finally, when affective commitment was the outcome of interest, 32% of variance was accounted for. Supervisor prosocial experiences accounted for over one-fifth of the variance (21%). Coworker prosocial experiences were the next strongest contributing factor at 12%. Supervisor (12%) and coworker (5%) incivility accounted for the remaining variance. Visualization of this breakdown can be found in Figure 1.

### 3.3. Follow-Up Analyses

Literature in the diversity, equity, and inclusion space suggests that subtle slights and prosocial experiences may be particularly meaningful to racial/ethnic minority group members. For instance, Cortina (2008) introduced the term selective incivility, which suggests that uncivil behaviors may be a modern-day form of discrimination. This was later empirically supported, finding that racial minority group members report significantly more experiences of incivility than their majority counterparts (Cortina et al., 2013). A similar construct of racial microaggressions has also emerged, which are subtle slights directed towards racial minority members (Solorzano et al., 2000). These experiences lead to negative psychological, emotional, and social well-being (e.g., Wong et al., 2013). This literature suggests that incivility experiences may be particularly relevant for shaping the job attitudes of minority workers, due to their increased frequency and repeated exposure. Conversely, microaffirmations are subtle acts that disrupt discrimination (e.g., Solis et al., 2024). Microaffirmations increase the sense of inclusion, value, and safety for targets (e.g., Solis et al., 2024). Therefore, prosocial experiences (e.g., providing genuine help and acknowledgment) may be particularly important in shaping job attitudes of minoritized workers by increasing feelings of inclusion.

**Figure 2:**
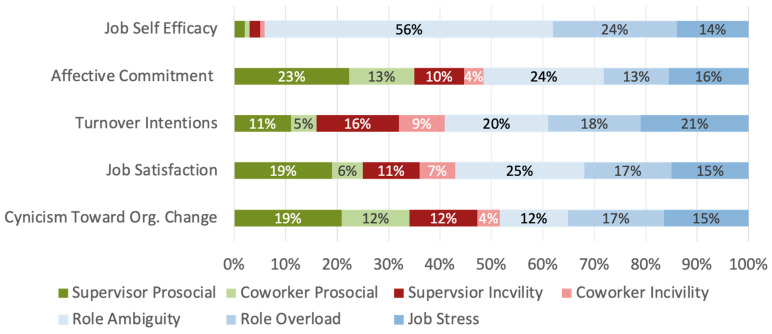
Visualization of Variance Breakdown for White Respondents.

**Figure 3:**
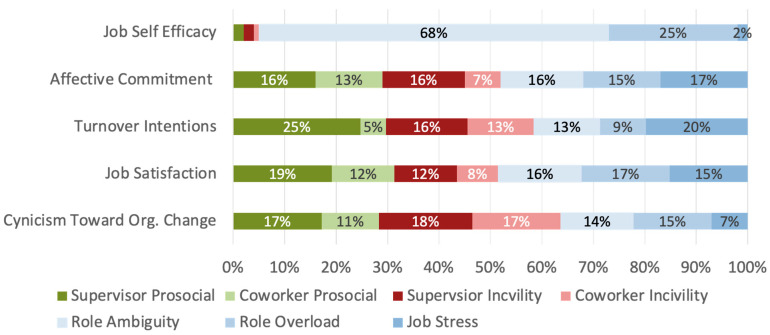
Visualization of Variance Breakdown for Non-White Respondents.

Based on these previous findings, follow-up analyses were conducted to examine how these results were influenced by whether the respondent belonged to a minoritized racial group or not (see Table 3 and Table 4 and Figure 2 and Figure 3). These results provide some initial support for the notion that interpersonal experiences differentially impact white and non-white workers’ job attitudes. First, independent samples *t*-tests were conducted to identify meaningful group differences in predictor variables. Common job stressors did not differ significantly across the two groups (job stress: t(1523) = 0.61, *p* = 0.54; role ambiguity: t(1576) = 0.09, *p* = 0.18; role overload: t(1568) = 0.25, *p* = 0.81). Further, supervisor (t(1543) = 1.63, *p* = 0.10) and coworker (t(1531) = 1.68, *p* = 0.09) prosocial experiences were only marginally significant across groups. Incivility experiences across both coworkers and supervisors demonstrated meaningful differences across groups. Specifically, non-white respondents (M = 0.84, SD = 0.85) indicated experiencing significantly more supervisor incivility than white respondents (M = 0.70, SD = 0.78), t(1557) = −2.43, *p* = 0.01. This same pattern existed for coworker incivility experiences (White: M = 0.75, SD = 0.74; Non-white: M = 0.88, SD = 0.76; t(1536) = −2.29, *p* = 0.02). Overall, these findings provide initial evidence that race is influencing interpersonal experience variables.

Supported by initial mean comparisons, relative weights analyses were conducted for white and non-white respondents separately. Taken together, interpersonal experiences accounted for more variance in job satisfaction (non-white 52%, white 43%), turnover intentions (non-white 59%, white 41%), and cynicism toward organizational change (non-white 63%, white 47%) for non-white employees.

When examining the specific patterns of the individual types of experiences, the starkest difference can be found in the impact of supervisor prosocial experience on turnover intentions. Supervisor prosocial experience accounted for 25% of the variance accounted for when examining non-white respondents and only 11% for white respondents. Additionally, coworker prosocial experiences accounted for a greater percentage of job satisfaction for non-white respondents, compared to white respondents, with 12% and 6% of variance accounted for, respectively. For organizational cynicism, coworker incivility accounted for a much larger proportion of variance for non-white respondents (17%), compared to white respondents (4%). Although the overall variance accounted for by interpersonal experiences did not differ between groups for affective commitment, the individual contributions of the different types of experiences did. Supervisor prosocial experiences accounted for 23% of the observed variance for non-white respondents and 16% for white respondents. For non-white respondents, incivility experiences accounted for a greater proportion of variance. Specifically, coworker incivility accounted for 3% more variance for non-white compared to white respondents (7% vs. 4%), and supervisor incivility accounted for 6% more variance (16% vs. 10%).

## 4. Discussion

### 4.1. Direct Effects of Prosocial and Incivility Experiences

This study identified that interpersonal experiences are significantly related to job attitudes. These findings replicate the work on incivility experiences by identifying significant relationships between incivility and affective commitment, intentions to leave the organization, and job satisfaction (Chris et al., 2022). Furthermore, this study was able to uniquely identify cynicism toward organizational change, having a significant relationship with incivility, an extension of previous work on general organizational cynicism (Manzoor et al., 2020).

Consistent with the broaden and build theory (Fredrickson, 2001), this study also identified that prosocial experiences have a significant relationship with job attitudes. This extends the current work on prosocial experiences by expanding the type of prosocial experiences (i.e., low-level positive interpersonal experiences) as well as expanding the type of outcomes examined. These findings are particularly timely due to the recent interest in applying positive psychology theories in the organizational literature. This provides evidence that these theories extend to workplace interpersonal experiences.

### 4.2. Co-Occurring Prosocial and Incivility Experiences

Positive and negative interpersonal experiences in the workplace do not occur in a vacuum. Because of this, a major contribution of this study was the simultaneous testing of the unique contributions of both incivility and prosocial experiences on job attitudes. Across all outcomes, about half of the variance accounted for in attitudes was attributed to interpersonal experiences, beyond that which was accounted for by the more typical stressors of role ambiguity, role overload, and job stress. This is especially impactful when considering how well-established the relationships between these typical stressors included in our model and job attitudes are. These findings provide support for affective events theory, which suggests that positive and negative experiences at work lead to affective responses, which shape subsequent job attitudes (Weiss & Cropanzano, 1996). Specifically, this study provides evidence for the co-occurrence of these positive and negative events and demonstrates how both types of experiences independently shape job attitudes. Practically, this study highlights that there is a need for organizations to create well-rounded surveys when considering interpersonal experiences in the workplace. The majority of measurement approaches for identifying interpersonal conflict of concerns in the workplace focus solely on mistreatment experiences; however, these findings suggest that it is equally critical to collect information about the prevalence or absence of prosocial interpersonal experiences to provide a clear picture of the day-to-day experiences of employees.

Further, our findings suggest that supervisor experiences play a much bigger role in shaping these attitudes than coworker experiences. This replicates the findings for workplace mistreatment (Hershcovis & Barling, 2010) and extends to prosocial experiences. This is further supported by the findings of Hitlan and Noel (2009), wherein organizational attitudes will be more strongly influenced by supervisor rather than coworker experiences. These findings suggest that supervisors’ behaviors are essential for shaping the working attitudes of their subordinates. Therefore, it is critical to properly train supervisors on effective interpersonal communication skills to prevent mistreatment and promote positive interactions.

### 4.3. Consideration of Stress as a Covariate

This study’s approach considered the unique contributions of interpersonal experiences above and beyond traditional work stressors. This study identified that about half of the variance accounted for by our model across job attitudes was influenced by well-established job stressors. While this provides important support for the role of interpersonal experiences in shaping job attitudes, it also highlights the importance of established job stressors in shaping these relationships. This may be particularly important when considering workplace mistreatment experiences where a major source of harm is a result of increased stress (Chris et al., 2022). Therefore, these findings suggest that it may be critical to include other sources of job stress as covariates in work on interpersonal experiences to provide clearer evidence of their true impacts on outcomes of interest, such as job attitudes and target well-being.

### 4.4. Examination of Race

Finally, follow-up analyses in this study explored how these relationships differed when examined based on racial status. Specifically, these data show that interpersonal experiences accounted for proportionally more variance in job satisfaction, turnover intentions, and organizational cynicism, but not affective commitment, for non-white employees. Additionally, across all outcomes, the specific contribution patterns of the different interpersonal experiences were meaningfully different. This provides initial evidence that the effects of interpersonal experiences at work are meaningfully different for white, compared to non-white workers.

These findings are supported within the diversity, equity, and inclusion literature in the form of related constructs of microaffirmations (i.e., subtle acts that disrupt discrimination) and microaggressions (i.e., low-intensity discrimination with unclear intent to harm; Solis et al., 2024). Specifically, racially marginalized workers experience more frequent incivility and other forms of mistreatment than their white counterparts (Cortina, 2008; Cortina et al., 2013), resulting in increased negative effects on their job attitudes due to repeated exposure. Further, prosocial acts, as defined in this research study, may act as microaffirmations for racial minority workers. Specifically, these behaviors can affirm the identities of marginalized people and increase feelings of belonging and inclusion (Rolón-Dow & Bailey, 2021). Therefore, promoting prosocial experiences and deterring incivility experiences may be particularly important for fostering a culture of diversity, equity, and inclusion. These positive and negative behaviors are not directly rooted in any marginalized identity. Because of this, this may be a fruitful strategy for increasing inclusion for marginalized employees without directly referring to any social identity. The lack of identity connection may be particularly relevant in the current social and political climate that is resisting Diversity, Equity, and Inclusion efforts, because it provides a space for organizations to engage in inclusion efforts without explicitly linking them to Diversity initiatives.

### 4.5. Limitations and Future Directions

This study is not without limitations. First, this study utilizes a university population to examine these relationships. While this included representation of a breadth of university positions, including both faculty and staff, there are questions of generalizability across other populations. For instance, when considering the supervisory roles of a university, staff positions may have a clear direct supervisor to whom they report regularly. However, faculty may perceive their supervisor as a Department Chair or Dean, depending on who each faculty member feels they report to. Additionally, many faculty members do not regularly report to their supervisor, as the position is highly autonomous. This may influence the regularity of interpersonal experiences and the potential effects on how workers view their job or organization. Therefore, future studies should aim to replicate these findings in different organizational settings.

Secondly, this study focused on organizational attitudes as the primary outcome variables of this study. Hitlan and Noel (2009) suggest that supervisor mistreatment influences organizational attitudes, and coworker experiences shape interpersonal attitudes. Therefore, the importance of supervisory attitudes may not extend to interpersonal attitudes, where coworker experiences may be a stronger driver. Future work should expand the outcomes of interest to include interpersonal outcomes as well as target well-being to identify the stability of these findings across a broader range of outcomes.

Further, this study utilized a single time point to collect all information. Because of this, no causal claims should be made. Although this study provides important evidence for the associations between interpersonal experiences and job attitudes, future work will have to establish the causal link between these constructs. Specifically, the use of longitudinal designs or daily diary studies would increase the ability to understand causal mechanisms between experiences and attitudes.

Due to the use of archival data, single-item measures were utilized to examine role overload and job stress in this study’s analyses. This may raise concerns that these items capture the breadth of the constructs. Therefore, future work should utilize validated multi-item measures of these constructs to ensure the relationships identified in this study are reliable across different forms of measurement. In a similar vein, although our sample supported a single factor for incivility experiences, it may be important to examine how the forms of uncivil behavior (e.g., incivility and exclusionary behavior) may have differential effects on job attitudes. For instance, within our sample, we found a significant, unexpected relationship between incivility and job self-efficacy. It may be the case that these associations are driven by the exclusion items, which may result in reduced access to resources needed to perform one’s job.

Finally, while our sample size was adequate to test the relationships of interest in this study, we were limited to operationalizing racial status as white and non-white for our follow-up analyses. This approach is not ideal because it equates all non-white racial identities as equal, even though there are vastly different social implications associated with different racial statuses in the United States context that may influence the experiences of both positive and negative experiences at work. Future research should strive for a sufficient sample size to test for unique effects among participants with various non-white racial identities.

Furthermore, there is a need to examine how intersectional identities may be transformative in the ways people experience mistreatment or prosocial behaviors. For example, stereotypes of black women are accompanied by unique forms of prejudice that black men and women of other races do not face. For instance, there is a stereotype of the “Strong Black Woman” where black women are perceived as resilient and not in need of support (e.g., Hall et al., 2025). This may manifest in fewer prosocial experiences or only being subject to compliments, independent of any meaningful support when needed. Additionally, the “Angry Black Woman” stereotype (e.g., Motro et al., 2022) may result in mistreatment experiences that include being dismissed when raising legitimate concerns, as well as uncivil behaviors like eye-rolling or making faces. Therefore, the manifestation of the experiences of both prosocial and incivility experiences may be shaped by the unique stereotypes that manifest from holding multiple marginalized identities, which, in turn, may influence the effects of these experiences. Therefore, future research should aim to examine intersectionality as a transformative mechanism for workers’ interpersonal experiences.

With acknowledgement of these limitations, this study was able to utilize unique statistical approaches to examine the simultaneous role of prosocial and incivility experiences in shaping job attitudes. This provides important theoretical insights into the lived experiences of workers’ everyday social relationships in the workplace. This opens additional areas for further exploration on this topic. First, future work should consider how experiencing prosocial and mistreatment experiences from the same individual shapes job attitudes or worker well-being. Under this line of inquiry, examination of how the proportion of different experiences shapes how people interpret those experiences may also be particularly fruitful. These concepts may also be extended to a team-level perspective. Specifically, aggregating experiences at a team level would allow researchers to understand how these collective experiences influence team functioning or performance. Additionally, researchers could examine the variation across the team in experiences to identify areas of social ostracism or manifestations of workplace bullying.

## 5. Conclusions

This study identified the importance of both incivility and prosocial experiences on workplace attitudes, including cynicism toward organizational change, job satisfaction, turnover intentions, and affective commitment. Findings suggest that prosocial work experiences and workplace incivility, in combination, account for similar variance as typical job stressors in employees’ job attitudes. Capturing both the positive and negative experiences that people have at work simultaneously represents a significant contribution to the workplace literature. Not only does this highlight the critical role interpersonal experiences play in shaping workplace attitudes, but it also suggests that examination of either in isolation does not provide a sufficient representation of employees’ workplace experiences. Additionally, supervisor experiences consistently accounted for more variance in outcomes than coworker experiences, highlighting the importance of well-trained supervisors. Finally, follow-up analyses provide preliminary support that these experiences are stronger drivers of aptitudes for non-white workers than white workers. This provides a meaningful area for diversity, equity, and inclusion interventions for organizations without needing to center marginalized identities, which may be particularly relevant under the current cultural push to move away from diversity, equity, and inclusion initiatives.

## Figures and Tables

**Table 1:** Correlations Among Study Variables.

	Mean (SD)	1	2	3	4	5	6	7	8	9	10	11	12
1. Supervisor incivility	0.73(0.79)	*α* = 0.87											
2. Coworker incivility	0.78(0.75)	0.47 ***	*α* = 0.86										
3. Supervisor prosocial behavior	2.64(1.00)	−0.63 ***	−0.26 ***	*α* = 0.94									
4. Coworker prosocial behavior	2.71(0.88)	−0.20 ***	−0.51 ***	0.51 ***	*α* = 0.91								
5. Cynicism toward org. change	2.63(1.35)	0.36 ***	0.28 ***	−0.41 ***	−0.32 ***	*α* = 0.90							
6. Job satisfaction	5.11(1.60)	−0.44 ***	−0.34 ***	0.50 ***	0.35 ***	−0.53 ***	-						
7. Turnover intentions	3.48(1.74)	0.39 ***	0.31 ***	−0.38 ***	−0.26 ***	0.43 ***	−0.63 ***	*α* = 0.85					
8. Affective commitment	4.97(1.42)	−0.26 ***	−0.26 ***	0.44 ***	0.34 ***	−0.62 ***	0.69 ***	−0.56 ***	*α* = 0.90				
9. Job stress	4.77(2.57)	0.27 ***	0.25 ***	−0.28 ***	−0.20 ***	0.33 ***	−0.43 ***	0.39 ***	−0.36 ***	-			
10. Role ambiguity	2.54(1.24)	0.38 ***	0.25 ***	−0.38 ***	−0.24 ***	0.38 ***	−0.50 ***	0.39 ***	−0.40 ***	0.33 ***	*α* = 0.83		
11. Role overload	3.16(1.76)	0.31 ***	0.25 ***	−0.32 ***	−0.21 ***	0.37 ***	−0.47 ***	0.37 ***	−0.36 ***	0.49 ***	0.42 ***	-	
12. Job self-efficacy	5.97(1.02)	−0.09 ***	−0.07 ***	0.11 ***	0.08 ***	−0.18 ***	0.24 ***	−0.11 ***	0.20 ***	−0.28 ***	−0.50 ***	−0.38 ***	*α* = 0.84

Note: *** *p* < 0.001; scale alpha values on the diagonal.

**Table 2:** Overall Relative Weights Results.

	Cynicism Toward Organizational Change		Job Satisfaction		Turnover Intentions		Affective Commitment		Job Self Efficacy	
	Relative Weight	Weight as a % of R^2^	Relative Weight	Weight as a % of R^2^	Relative Weight	Weight as a % of R^2^	Relative Weight	Weight as a % of R^2^	Relative Weight	Weight as a % of R^2^
Role ambiguity	0.055	19.23	0.100	22.29	0.056	18.46	0.065	20.63	0.190	59.31
Role overload	0.048	16.84	0.078	17.44	0.048	15.72	0.041	13.17	0.079	24.64
Job stress	0.036	12.73	0.067	14.95	0.063	20.79	0.049	15.56	0.033	10.46
Supervisor prosocial	0.054	19.02	0.086	19.08	0.042	13.73	0.067	21.41	0.006	1.95
Coworker prosocial	0.034	12.05	0.034	7.50	0.017	5.57	0.041	13.09	0.003	0.78
Supervisor incivility	0.039	13.63	0.053	11.72	0.049	16.10	0.036	11.63	0.007	2.09
Coworker incivility	0.019	6.51	0.032	7.02	0.029	9.64	0.014	4.51	0.003	0.77
Total R^2^	0.29		0.45		0.30		0.32		0.32	

**Table 3:** Relative Weights Results for White Respondents.

	Cynicism Toward Organizational Change		Job Satisfaction		Turnover Intentions		Affective Commitment		Job Self Efficacy	
	Relative Weight	Weight as a % of R^2^	Relative Weight	Weight as a % of R^2^	Relative Weight	Weight as a % of R^2^	Relative Weight	Weight as a % of R^2^	Relative Weight	Weight as a % of R^2^
Role ambiguity	0.061	12.42	0.115	24.69	0.064	20.32	0.072	23.61	0.179	55.80
Role overload	0.047	16.50	0.087	17.38	0.056	17.79	0.041	13.17	0.078	24.33
Job stress	0.041	14.61	0.070	15.10	0.066	20.94	0.048	15.52	0.044	13.85
Supervisor prosocial	0.053	18.89	0.087	18.70	0.036	11.37	0.069	22.58	0.006	1.90
Coworker prosocial	0.034	12.08	0.030	6.36	0.017	5.42	0.038	12.50	0.003	0.93
Supervisor incivility	0.034	12.08	0.052	11.25	0.049	15.63	0.030	9.92	0.007	2.28
Coworker incivility	0.013	4.42	0.030	6.53	0.027	8.53	0.011	3.65	0.003	0.91
Total R^2^	0.28		0.46		0.32		0.31		0.31	

**Table 4:** Relative Weights Results for Non-White Respondents.

	Cynicism Toward Organizational Change		Job Satisfaction		Turnover Intentions		Affective Commitment		Job Self Efficacy	
	Relative Weight	Weight as a % of R^2^	Relative Weight	Weight as a % of R^2^	Relative Weight	Weight as a % of R^2^	Relative Weight	Weight as a % of R^2^	Relative Weight	Weight as a % of R^2^
Role ambiguity	0.045	13.8	0.065	15.76	0.036	13.05	0.057	16.15	0.221	67.96
Role overload	0.050	15.46	0.071	17.23	0.023	8.55	0.055	15.41	0.080	24.64
Job stress	0.024	7.43	0.060	14.61	0.053	19.58	0.059	16.61	0.008	2.35
Supervisor prosocial	0.056	17.47	0.080	19.49	0.068	25.13	0.057	16.19	0.007	2.17
Coworker prosocial	0.036	11.09	0.050	12.20	0.014	5.26	0.047	13.35	0.002	0.45
Supervisor incivility	0.057	17.67	0.050	12.29	0.043	15.63	0.056	15.67	0.005	1.63
Coworker incivility	0.055	17.09	0.035	8.42	0.035	12.80	0.023	6.62	0.003	0.80
Total R^2^	0.31		0.41		0.27		0.35		0.32	

## Data Availability

The data presented in this study are available on request from the corresponding author due to participant data protection and storage requirements of the institutional review board.
